# Necrotizing Herpetic Retinopathy: A Case of Delayed Acute Retinal Necrosis Presentation in a Patient With Uncontrolled Diabetes Mellitus and Complications

**DOI:** 10.7759/cureus.93601

**Published:** 2025-09-30

**Authors:** Jacklyn Vainshtein, Dagny Zhu, Moisés Enghelberg, Michael Eden, Amanda Frugoli

**Affiliations:** 1 Medicine, Western University of Health Sciences, Pomona, USA; 2 Ophthalmology, NVISION Eye Center, Rowland Heights, USA; 3 Ophthalmology, Dougherty Laser Vision, Ventura, USA; 4 Internal Medicine, Community Memorial Healthcare, Ventura, USA; 5 Medical Educatiuon, Community Memorial Healthcare, Ventura, USA

**Keywords:** acute retinal necrosis, delayed acute retinal necrosis, intravitreal ganciclovir, necrotizing herpetic retinopathy, painless vision loss, retinal detachment (rd), smoking tobacco, uncontrolled diabetes mellitus type 2

## Abstract

Necrotizing herpetic retinopathy is a term used to describe two similar yet different forms of retinopathy - acute retinal necrosis (ARN) and progressive outer retinal necrosis (PORN), which can both present similarly with eye pain and/or vision loss stemming from inflammatory and necrotic retinal changes caused by members of the *Herpesviridae* family. This case presentation involves a patient with ARN, which has a multifocal pathogenesis, creating diffuse necrosis that over time coalesces and increases the risk of rhegmatous retinal detachment.

We report the case of a 56-year-old male with uncontrolled insulin-dependent diabetes mellitus complicated by retinopathy and heavy tobacco use who presented with nonspecific symptoms of left eye redness, burning ocular pain, and blurred vision with lateral field vision loss for two weeks. Thorough dilated fundus examination with retinal imaging identified 360-degree retinal necrosis extending to the periphery, mild retinal hemorrhages, and moderate disk edema. Serology was found to be positive for herpes simplex virus (HSV)-1 IgG, which did not confirm ongoing infection as HSV-1 IgM was negative. Other testing, including HSV-2 IgG, human immunodeficiency virus (HIV)-1 and HIV-2 screening antibody, QuantiFERON Gold (Qiagen, Hilden, Germany), cytomegalovirus (CMV) IgG and IgM, and polymerase chain reaction (PCR) for toxoplasmosis, was negative. Intravitreal fluid sampling was negative for HSV-1 and 2 DNA by PCR, although CMV and varicella zoster virus (VZV) were not tested due to lab limitations. The patient was placed on standard ARN treatment with IV acyclovir three times a day, ophthalmic prednisolone drops two to four times a day, and a single dose of intravitreal ganciclovir injection. At the time of discharge, associated symptoms had resolved with minimal vision improvement. Follow-up was complicated by a total retinal detachment leading to complete loss of vision in the left eye.

Due to complicated compounding factors found in our patient, many factors may have influenced his severe complications and poor prognosis following discharge, despite appropriate treatment. It has been shown that uncontrolled diabetes can make individuals immunocompromised and predispose them to many infectious diseases. It is possible that this could be a risk factor for our patient. Although ARN is associated with retinal detachment, we are not able to exclude underlying diabetic retinopathy and vascular atherosclerosis related to tobacco dependence as contributing factors. Although there has been some evidence of a commonality between ARN and patients with pre-existing diabetes mellitus, there is no current research found to describe the interplay between uncontrolled diabetes with diabetic retinopathy, continued tobacco use, and the sequential development of ARN.

This case report discusses a patient presenting with classic symptoms of a unique retinal pathology, ARN, complicated by severe retinal detachment. The goal of this case report is to bring awareness of a rare disease to assist providers in making an efficient diagnosis to prevent vision loss.

## Introduction

Necrotizing herpetic retinopathy is a term used to describe two similar yet different forms of retinopathy - acute retinal necrosis (ARN) and progressive outer retinal necrosis (PORN) [1]. Both of these can present similarly with eye pain and/or vision loss stemming from inflammatory and necrotic retinal changes caused by members of the herpesviridae family 1, including varicella zoster virus (VZV), herpes simplex virus (HSV)-1 and HSV-2, and cytomegalovirus (CMV) [2].

Differentiating between ARN and PORN is important for administering treatment and gauging long-term complications. Delayed treatment for any type of necrotizing herpetic retinopathy can result in permanent vision loss due to retinal detachment. One main difference is that ARN presents more commonly in patients who are immunocompetent, while PORN presents in patients who are immunocompromised, with relative immunosuppression being defined as a CD4 count <50 [3,4]. In addition, the multifocal whitened necrotic lesions in ARN typically begin in the peripheral retina with ARN, whereas PORN can directly involve the posterior pole in early stages. Retinal vasculitis, vitritis, and anterior chamber reactions have all been found to be associated with ARN, but are less common with PORN [4,5].

The pathogenesis of ARN is multifocal, creating diffuse necrosis, which over time coalesces and increases the risk of rhegmatous retinal detachment. This occurs in multiple phases, with the first phase presenting predominantly as an anterior chamber reaction with varying degrees of vitritis and deep retinal infiltrates seen peripherally on dilated fundoscopy [6]. With disease progression, the infiltrative retinal lesions necrose and spread towards the posterior pole, where central vision can be affected. Late in disease progression, the retina atrophies, and vitreal traction from ongoing vitreous inflammation induces retinal detachments. All of these phases progress rapidly, with permanent vision loss being the most feared outcome [6].

The histology of ARN is unique in that retinal cells present with eosinophilic intranuclear inclusions along with retinal necrosis and arteritis affecting all layers of the associated retinal portions [2].

On presentation, a patient will present with a complaint of rapid-onset pain, with or without redness and blurry vision. On dilated fundus examination, a triad of 1) vascular arteritis, 2) peripheral necrotizing retinitis, and 3) vitritis is usually found [2].

This case report discusses a patient who presented with an atypical presentation of ARN confounded by diabetic retinopathy. The goal of this discussion is to provide an example of a rare disease with delayed diagnosis in the setting of underlying diabetic retinopathy and poor surgical candidacy, in order to assist other care providers with making an efficient diagnosis. It also demonstrates the continued importance of competence in and completion of retinal examinations.

This case and abstract were presented as a poster presentation at the Association for Research in Vision and Ophthalmology Annual Meeting in 2025. The abstract was published in the ARVO journal.

## Case presentation

A 56-year-old man was directly admitted to the hospital upon the request of his ophthalmologist, who had been evaluating the patient for left eye redness, burning pain, blurred vision, and lateral field vision loss over a period of two to three weeks. The initial evaluation included a dilated fundoscopic examination, retinal angiography, and a CT scan of the patient's brain and orbits. Fundoscopic examination of the left eye was positive for optic nerve edema, diffuse retinal hemorrhage, and areas of retinal whitening with vasculitis. Additionally, some of the imaging was blurry due to ongoing vitritis. MRI was not performed as the patient's pacemaker was incompatible. 

His medical history included hypertension, insulin-dependent type II diabetes complicated by retinopathy, heavy tobacco use, atrial fibrillation on Xarelto, sick sinus syndrome with a pacemaker, and chronic lower extremity edema.

**Figure 1:**
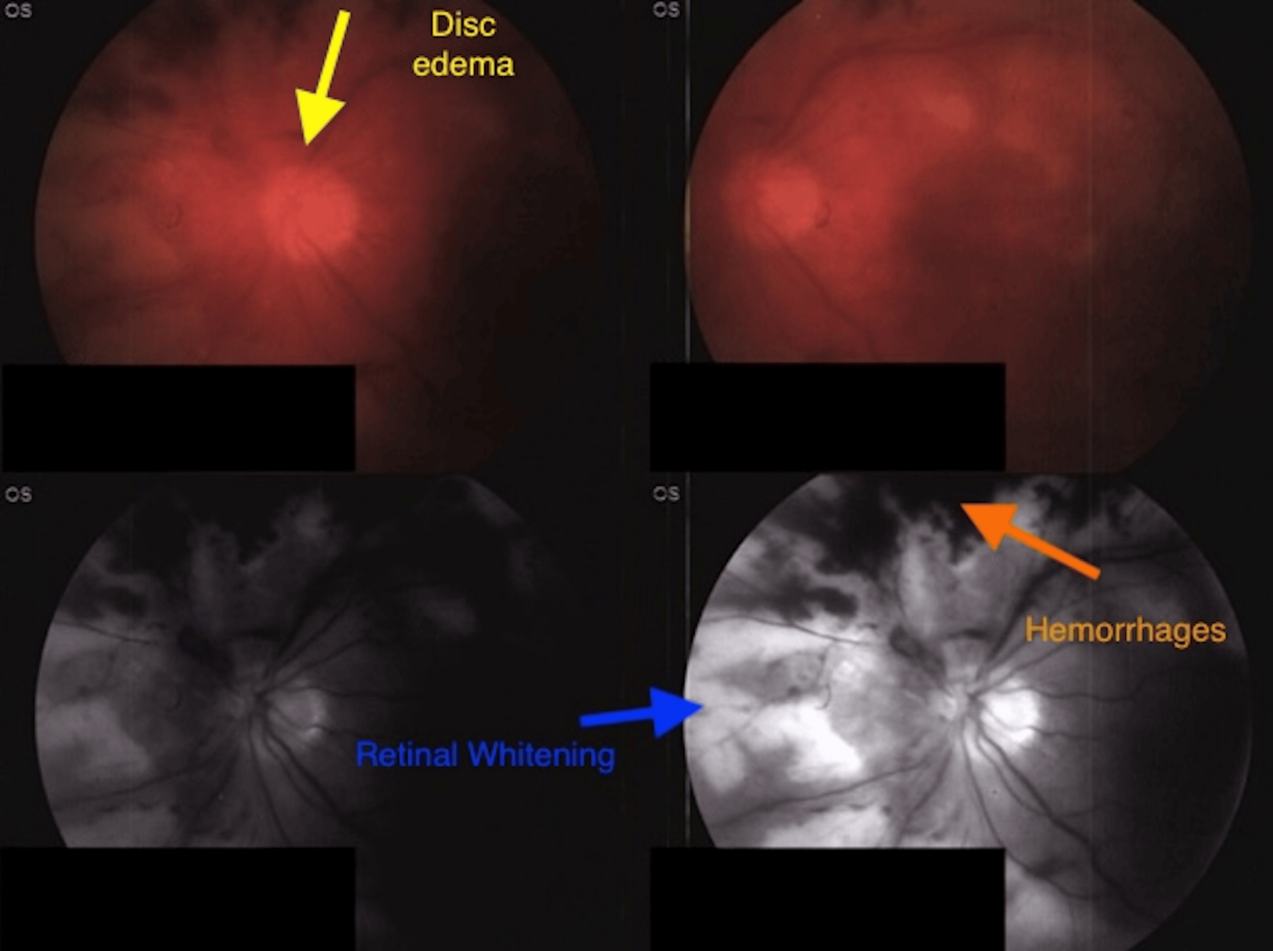
Dilated fundus exam demonstrating classic ARN findings including hemorrhages (orange), retinal whitening (blue) and disc edema (yellow)

Upon admission to the hospital, the patient reported that his symptoms had progressively worsened over two weeks, and he also reported an associated nausea and diffuse headache. A dilated fundus exam was performed, demonstrating retinal necrosis extending from the periphery 360 degrees to the vascular arcades, mild retinal hemorrhage, moderate disk edema with vitreal hemorrhage. Retinal detachment was not noted upon initial imaging (Figure 1). 

The patient was started presumptively on weight-based intravenous acyclovir three times a day and was given one intravitreal injection of ganciclovir. He was also started on ophthalmic prednisolone, one drop in each eye four times a day. At the same time, additional testing was undertaken to evaluate for viral and bacterial etiologies (Table 1).

**Table 1:** Infectious etiology results HSV - herpes simplex virus; HIV - human immunodeficiency virus; CMV - cytomegalovirus

Test	Result	Reference
Serum HSV-1 IgG	41.1	<0.9 = Negative
Serum HSV-2 IgG	<0.9	<0.9 = Negative
Serum HIV-1 and HIV-2 screening antibody	Non-reactive	Non-reactive
Serum QuantiFERON Gold	Negative	Negative
Serum CMV IgG	<0.60 U/mL	<0.60 U/mL
Serum CMV IgM	<30.00 AU/mL	<30.00 AU/mL
Serum toxoplasmosis	Not detected	Not detected
Intravitreal HSV-1	Not detected	Not detected
Intravitreal HSV-2	Not detected	Not detected

At the time of discharge, the patients' symptoms of pain and redness had improved with decreased shadowing in the upper visual field, though minimal improvement in left eye visual acuity was noted. The patient was discharged after three days in the hospital and continued on prednisolone eye drops and intravenous acyclovir for one week, with reevaluation by infectious disease and ophthalmology. 

His follow-up was complicated by unilateral retinal detachment with complete loss of vision in his left eye. No surgical intervention was pursued given the severity of the detachment, the lack of perceived surgical benefit, and the patient's preference. The patient was lost to follow up and serial dilated fundus exams were not acquired.

## Discussion

Due to the compounding factors found in our patient, it is important to focus on each as a potential influencing factor in the severe progression and ultimately poor prognosis of his ARN. 

Immunocompetence is the main differentiating factor between ARN and PORN. It has been defined that a patient with PORN is considered immunocompromised if their CD4 count is <50. Although our patient had no evidence of HIV induced immunocompromisation, this patient likely had a subclinical immunocompromised state due to chronic uncontrolled type 2 diabetes mellitus and its associated hyperglycemia. Chronically elevated levels of glucose over 200 have been found to induce an acidotic state in patients with diabetes, which in turn reduces their immune system by impairing neutrophil chemotaxis, creating a relative immunocompromised state [7]. Given our patient's additional history of diabetic retinopathy, we cannot state with certainty whether it was the acute retinal necrosis or other underlying retinal causes that triggered his ultimate retinal detachment. 

Since ARN is described as an inflammatory process, the patient's smoking history can also be implicated as an exacerbating factor, though no direct correlation has been investigated. Tobacco has been found to stimulate an inflammatory and angiogenic reaction in retinal cells [8]. Excessive nitric oxide levels produced by tobacco have been found to cause oxidative stress, which has been implicated in the contribution of vascular damage found in retinopathies [8]. Significant associations have also been found between smoking and posterior retinal detachment, implying that it could have played a role in the retinal detachment that was seen in our patient [9].

Additionally, our patient was compared to a case series report where four out of 22 patients diagnosed with ARN were found to also have diabetes mellitus [10]. Of those four patients, two also presented with complications of retinal detachment, like our patient [10]. However, out of these four patients, three presented with VZV as the causative organism while the fourth's was unknown. Out of all the other patients in the study, the only other pre-existing condition that showed up more consistently than diabetes was Herpes zoster ophthalmicus [10]. It was also found that the male gender was associated with worse disease prognosis and an association with retinal detachment, as seen with our patient as well [10]. Our patient, however, only tested positive for HSV IgG serology, implying that at some point he may have been infected with HSV-1. In the presence of negative intravitreal PCR for HSV-1 and HSV-2, there is no validation that HSV could have been the cause of his ARN. Other etiologies of ARN could have been VZV or CMV, but this patient's intravitreal sample was not tested for these two etiologies due to hospital limitations and the desire for a rapid test result. Based on the dilated fundus exam and presence of unilateral symptoms, it is speculated that VZV was the underlying cause, although he had no history of VZV, and no pathognomonic skin lesions were identified. 

Our patient was placed on the gold standard ARN treatment consisting of IV acyclovir three times a day, one intravitreal injection of ganciclovir, and one drop of ophthalmic prednisolone in the left eye four times daily, started upon admission. Systemic antivirals, specifically IV acyclovir, have been found to help decrease involvement of the optic nerve and prevent transfer to the other eye [11]. An IV acyclovir is preferred because of its rapid onset, especially in patients with severe and sudden symptoms that need rapid treatment [6]. The intravitreal ganciclovir injection was administered for our patient on day two of his hospital stay due to the hospital pharmacy not having it in stock, and it needed to be injected by the ophthalmologist. Intravitreal injections have been shown to help prevent retinal detachment [12]. Prednisolone drops were administered in an attempt to decrease inflammation. A topical steroid drop was chosen due to the patient's history of uncontrolled diabetes. 

Ultimately, our patient's case resulted in a unilateral retinal detachment on the side affected by ARN. Retinal detachment is common in patients with ARN, happening in about 50-75% of cases. In our patient's case, the pathogenesis of retinal detachment was likely due to a combination of ARN itself, poor healing due to diabetes and tobacco use, and delayed presentation time. Our patient presented to his primary care physician with complaints of pain, redness, and vision loss, two to three weeks before he was eventually sent to the hospital for treatment. In this case, it is possible that earlier treatment could have decreased his risk of retinal detachment. 

## Conclusions

In conclusion, many confounding factors played important roles in the pathogenesis and ultimate outcome of this patient's disease course. It is of utmost importance for medical providers to understand the disease process and presentation of diseases like necrotizing herpetic retinopathies so that they are able to efficiently diagnose with appropriate testing and treat these patients to prevent long-term complications. Urgent dilated ophthalmologic exams by an ophthalmologist are of utmost importance in diabetic patients with new visual field changes or sudden loss of vision.
